# Changes in smoking, alcohol consumption, and the risk of Parkinson’s disease

**DOI:** 10.3389/fnagi.2023.1223310

**Published:** 2023-09-13

**Authors:** Se Young Jung, Sohyun Chun, Eun Bin Cho, Kyungdo Han, Juhwan Yoo, Yohwan Yeo, Jung Eun Yoo, Su Min Jeong, Ju-Hong Min, Dong Wook Shin

**Affiliations:** ^1^Department of Family Medicine, Seoul National University Bundang Hospital, Seongnam, Republic of Korea; ^2^Department of Digital Healthcare, Seoul National University Bundang Hospital, Seongnam, Republic of Korea; ^3^International Healthcare Center, Samsung Medical Center, Seoul, Republic of Korea; ^4^Department of Neurology, College of Medicine, Gyeongsang Institute of Health Science, Gyeongsang National University, Jinju, Republic of Korea; ^5^Department of Statistics and Actuarial Science, Soongsil University, Seoul, Republic of Korea; ^6^Department of Biostatistics, The Catholic University of Korea, Seoul, Republic of Korea; ^7^Department of Family Medicine/Supportive Care Center, Samsung Medical Center, Sungkyunkwan University School of Medicine, Seoul, Republic of Korea; ^8^Department of Family Medicine, Healthcare System Gangnam Center, Seoul National University Hospital, Seoul, Republic of Korea; ^9^Department of Neurology, Samsung Medical Center, Sungkyunkwan University School of Medicine, Seoul, Republic of Korea; ^10^Neuroscience Center, Samsung Medical Center, Seoul, Republic of Korea; ^11^Department of Health Sciences and Technology, Samsung Advanced Institute for Health Sciences and Technology (SAIHST), Sungkyunkwan University, Seoul, Republic of Korea; ^12^Department of Clinical Study Design and Evaluation, Samsung Advanced Institute of Health Science and Technology (SAIHST), Sungkyunkwan University, Seoul, Republic of Korea; ^13^Center for Wireless and Population Health Systems, University of California, San Diego, La Jolla, CA, United States

**Keywords:** cigarette smoking, alcohol consumption, Parkinson’s disease, Asia, nicotine

## Abstract

**Objective:**

There have been no studies on the association between changes in smoking and alcohol consumption or combined changes in smoking and alcohol consumption frequencies and PD risk. To assess the influence of changes in smoking and alcohol consumption on the risk of Parkinson’s disease (PD).

**Methods:**

National Health Insurance Service (NHIS) database between January 2009 to December 2011 was analyzed. A total of 3,931,741 patients were included. Study participants were followed up for the incidence of PD until December 2017.

**Results:**

Compared to the sustained non-smokers, sustained light smokers (adjusted hazard ratio [aHR] 0.80, 95% confidence interval [CI] 0.75–0.85), sustained moderate smokers (aHR 0.54, 95% CI 0.47–0.61), and sustained heavy smokers (aHR 0.49, 95% CI 0.44–0.55) had a lower risk of PD. Compared to those who sustained non-drinking, sustained light drinkers (aHR 0.85 95% CI 0.89–0.91), sustained moderate drinkers (aHR 0.68, 95% CI 0.60–0.78), and sustained heavy drinkers (aHR 0.77, 95% CI 0.68–0.87) showed decreased risk of PD. Among non-drinkers, those who started drinking to a light level were at decreased risk of PD (aHR 0.84, 95% CI 0.77–0.91). Among non-smoking and non-drinking participants, those who initiated smoking only (aHR 0.78, 95% CI 0.70–0.86), drinking only (aHR 0.77, 95% CI 0.68–0.87), and both smoking and drinking (aHR 0.69, 95% CI 0.58–0.82) showed decreased risk of PD.

**Conclusion:**

Smoking is associated with decreased risk of PD with a dose–response relationship. Alcohol consumption at a light level may also be associated with decreased risk of PD. Further studies are warranted to find the possible mechanisms for the protective effects of smoking and drinking on PD, which may present insights into the etiology of PD.

## Introduction

Neurological diseases are the leading cause of disability worldwide (GBD 2016 Neurology Collaborators, 2019), and the prevalence of Parkinson’s disease (PD) is increasing more rapidly than other neurological diseases (Ou et al., 2021). Based on a systematic analysis of epidemiological studies, the worldwide prevalence of PD increased from 2.5 million in 1990 to 6.1 million in 2016 (GBD 2016 Parkinson's Disease Collaborators, 2018). Another study conducted in South Korea found that the incidence of PD continuously increased between 2010 and 2015 (Park et al., 2019a). Previous epidemiologic studies have provided important clues to potential risk factors related to PD. Data for most putative risk factors for PD are conflicting; however, consistent evidence was obtained suggesting that a family history of PD and older age are associated with an increased risk of PD, while smoking is associated with a decreased risk (Noyce et al., 2012).

Regarding smoking, previous studies have repeatedly reported a decreased risk association with PD. A previous meta-analysis of 61 case–control and 8 cohort studies reported that current smoking was associated with a 58% risk reduction of PD (relative risk [RR] 0.42; 95% confidence interval [CI] 0.38–0.47) (Li et al., 2015). Furthermore, a prospective cohort study by Mappin-Kasirer et al. (2020) concluded that smoking is casually associated with a decreased risk of PD. In that research, the authors tracked changes in smoking habits by surveying 30,000 British doctors seven times over 65 years from 1951 to 1998 and found that smokers had a 40% decreased risk of PD compared with never-smokers (RR 0.60, 95% CI 0.46–0.77) (Mappin-Kasirer et al., 2020). In addition, the group found that duration since quitting was also related to the risk of developing PD (RR 0.71, 95% CI 0.54–0.93) for those who quit smoking less than 9 years previously. The RR was 0.86, 95% CI 0.70–1.06 for those who quit smoking 10 or more years previously. The authors concluded that this is evidence for a causal protective effect of current smoking on the risk of developing PD. In addition, a recent Mendelian randomization study reported that smoking is a protective factor against PD when comparing current to former smokers (odds ratio [OR] 0.64, 95% CI 0.46–0.89) (Supplementary Table S1; Dominguez-Baleon et al., 2021). Since smoking may have a protective effect against PD, and smoking frequency worldwide has been decreasing since the mid-2000s, this may explain the rising incidence of PD (Elflein, 2022).

However, previous studies on the association between alcohol and risk of PD had conflicting results. A meta-analysis, in which eleven prospective studies were included, suggested that alcohol consumption was related with a decreased risk of PD (RR 0.81, 95% CI 0.70–0.95) (Shao et al., 2021). The above-mentioned Mendelian randomization study reported increased alcohol consumption had a protective effect against PD (OR 0.79, 95% CI 0.65–0.96) (Dominguez-Baleon et al., 2021). However, a multi-national cohort study of 521,000 participants in Europe reported that there were no associations between baseline or lifetime total alcohol consumption and the risk of PD (Peters et al., 2020). Another cohort study of 1,309,267 women conducted in South Korea reported that there was no significant trend in alcohol-associated PD development risk among never smokers (Kim et al., 2020b). Decreased smoking frequency may affect increased PD incidence, and alcohol consumption also may influence the decreased incidence of PD because global alcohol consumption has decreased since 1980 (Hannah Ritchie, 2018).

While numerous studies have explored the relationship between baseline frequencies of cigarette smoking and alcohol consumption with the risk of Parkinson’s Disease (PD), whether changes in these habits are associated with the risk of PD is rarely discussed. Understanding how modifying smoking and alcohol drinking habits can influence the risk of PD, especially when considering their combined effects, could help illuminate the potential link between reduced intake of both substances and an increased PD incidence. Given the sparce research on the influence of changes in consumption over time, our study aimed to examine how alterations in smoking and alcohol consumption—both separately and in combination—can affect the risk of Parkinson’s disease. To achieve this, we utilized a comprehensive dataset from the National Health Insurance Service (NHIS) of South Korea.

## Methods

### Data source and study setting

This research used the NHIS database. The NHIS is the single government health insurer in South Korea. NHIS has covered approximately 97% of the South Korean population since being launched as the compulsory health insurance program in 1997 (Lee et al., 2016). The Medical Assistance Program covers the remaining 3% of the population with low income.

The NHIS database includes reimbursement claims submitted by healthcare providers. This database also has data on demographics, medications, procedures, and disease diagnoses based on the International Classification of Disease, 10th revision (ICD-10). In addition, the NHIS supports biennial national health examination information. This information consists of anthropometric measurements, laboratory test results, and self-reported questionnaires. The questionnaire provides information on patient smoking frequency, alcohol consumption, and exercise programs. The detailed cohort profile of the NHIS database has been provided in a previous study (Cheol Seong et al., 2017).

**Figure 1:**
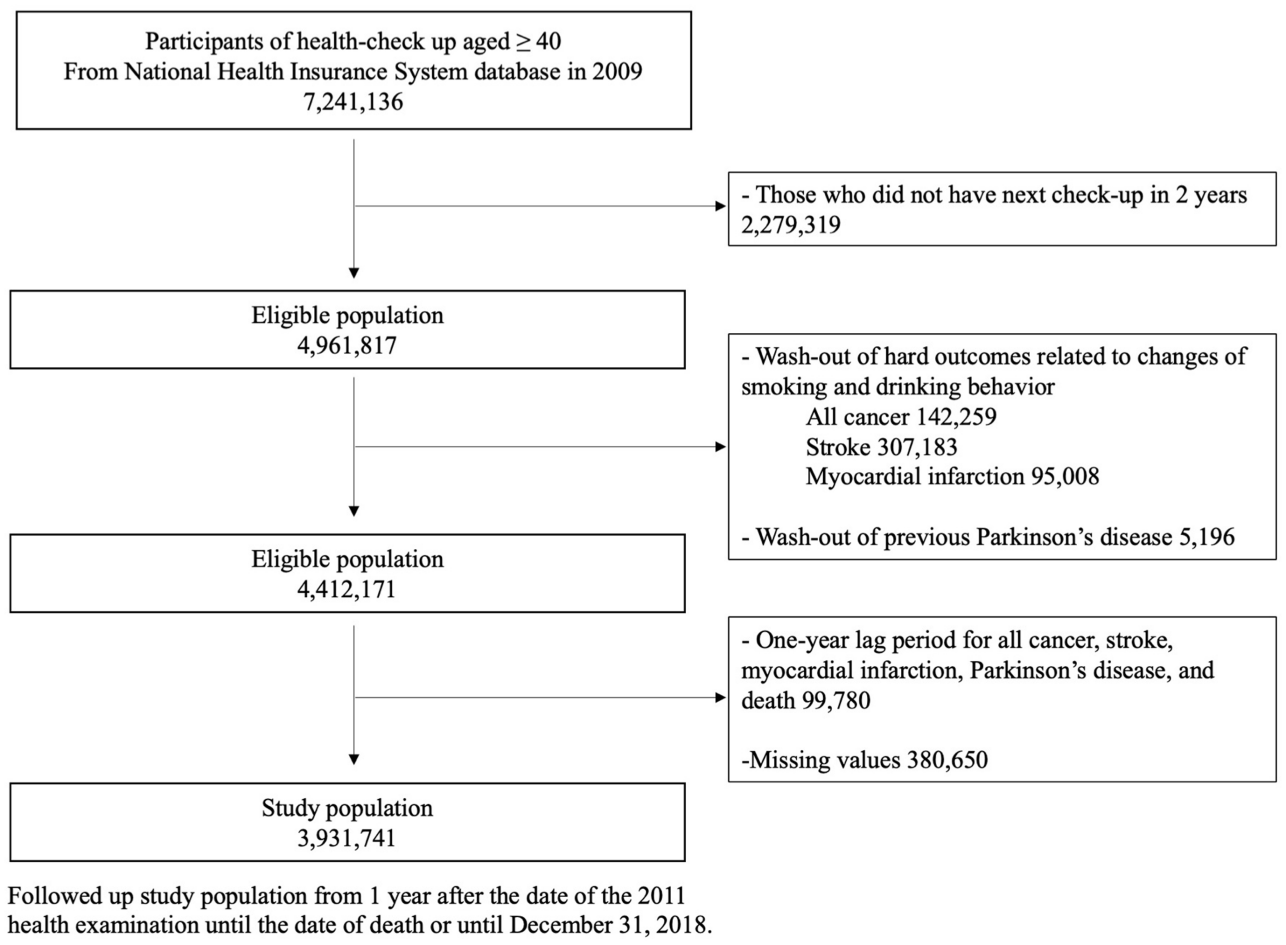
Flow chart of study participants.

We extracted data on participants who were 40 years old or older and underwent a general health examination in 2009 (*n* = 7,241,136). To determine changes in smoking frequency and alcohol consumption, those who had a follow-up examination in 2011 were included as study participants (*n* = 4,961,817). Since smoking and alcohol consumption are significant risk factors for cancer, myocardial infarction, and stroke, and because these conditions might affect one’s smoking and drinking habits, we excluded participants who (1) had a history of cancer (*n* = 142,259), myocardial infarction (*n* = 95,008), or stroke (*n* = 307,183) prior to the index date (Total *n* = 549,646) and (2) were diagnosed with cancer, stroke, myocardial infarction, PD, or had a recorded death within 1 year from the index date (*n* = 99,780). We also excluded who had missing data for at least one variable (*n* = 380,650). Since smoking frequency, alcohol consumption, and exercise information are self-reported, those who did not respond to any of these questions were also excluded from the study cohort. A total of 3,931,741 participants were selected for the final study population. This group was followed from 1 year after the date of the 2011 health examination until the date of death or until December 31, 2018 (Figure 1).

### Definition of cigarette smoking behaviors and alcohol consumption

#### Cigarette smoking behaviors

The questionnaire first asked, ‘Have you smoked at least 100 cigarettes in your entire life?’. Those who answered ‘no’ were considered non-smokers, and the rest were considered ever-smokers. Among ever-smokers, those who checked at the statement of ‘I am currently smoking.’ were considered current smokers and others were regarded as past smokers. For current smokers, information on average daily smoking frequency and duration of smoking were collected: (1) light smokers (< 10 cigarettes per day), (2) moderate smokers (10–19 cigarettes per day), and (3) heavy smokers (≥ 20 cigarettes per day). Past smokers were categorized into the same group based on their average daily smoking frequency and duration of smoking at the time they were smoking.

#### Alcohol consumption

Participants were asked about their total weekly alcohol consumption. The weekly alcohol consumption was calculated by multiplying the alcohol consumption frequency and amount per drinking occasion. We classified the amount of alcohol consumption into four categories: (1) none, (2) light drinker (less than 15 g/day on average), (3) moderate drinker (more than 15 g/day but less than 30 g/day), and (4) heavy drinker (more than 30 g/day) (Yoo et al., 2021).

### Study outcomes and follow-up

The primary end point was newly diagnosed PD during the follow-up period. Newly diagnosed PD was defined by the ICD-10 code (G20 for Parkinson’s disease) and the registration code (V124) for PD. The Korean government has operated a registration program for rare intractable diseases (RID) since 2006. The enrollees of the program can reduce copayment by up to 10%, as compared to 20–30% for general medical care. Therefore, almost all PD patients are expected to be registered to this program. The inclusion criteria for V124 are based on parkinsonism-related symptoms but exclude secondary causes such as stroke or head injury. To participate in this program, a doctor, typically a neurologist or primary care physician, certifies the PD diagnosis in writing based on the clinical criteria (Park et al., 2019b). In S. Korea, the diagnosis of PD primarily follows the UK criteria, ensuring high accuracy (Kalia and Lang, 2015; Park et al., 2019a; Kang et al., 2023). Study participants were followed from the date of entry to the earliest date of PD diagnosis, the end date of the study (December 31, 2018), or the end of follow-up, whichever came first.

### Covariates

Several variables were included as covariates: age, sex, body mass index (BMI), and presence of diabetes mellitus or dyslipidemia. Diabetes and dyslipidemia were found to be related to the risk of PD in previous studies (Jeong et al., 2019, 2020). Diabetes mellitus was defined by ICD-10 codes E10 through E14 with at least one prescription of an anti-diabetic medication or a fasting glucose level of 126 mg/dL or more, and dyslipidemia was defined by ICD-10 code E78 with at least one prescription of a lipid-lowering drug or a total cholesterol level of 240 mg/dL or more.

### Statistical analysis

Continuous variables and categorical variables are presented as means ± standard deviations (SDs) and numbers (percentages), respectively, for descriptive statistics. For the baseline characteristics of the study participants, continuous variables were compared between the two groups using Student’s *t*-tests, while categorical variables were assessed using either the *χ*^2^ test or the Fisher exact test.

The incidence of PD associated with changes in smoking and alcohol consumption habits was estimated by Cox proportional hazards regression analysis using a crude and multivariable-adjusted model. Final multivariate model was adjusted for age, sex, income, body mass index, and drinking status at baseline, diabetes mellitus, and dyslipidemia. Hazard ratios (HRs)were considered significantly different from the reference when 95% CI did not include 1. Hazard ratios (HRs) and 95% CIs were calculated. The comparisons were performed using two separate reference groups: sustained non-drinkers or non-smokers were used to compare the association across all categories, while sustainers (e.g., light–light) were used to compare the association within each baseline category. All statistical analyses were performed using SAS statistical package version 9.4 (SAS Institute Inc., Cary, NC, USA), and a value of *p* <0.05 was considered statistically significant.

## Results

During the mean follow-up period of 6.34 ± 0.62 years, 12,642 individuals were diagnosed with PD. PD patients were older (66.10 ± 8.80 years in the PD group vs. 55.02 ± 9.64 years in the non-PD group), and the PD group had a lower proportion of male subjects (48.99% in the PD group vs. 51.63% in the non-PD group) (Table 1). Additionally, there were fewer patients who reported being current smokers (9.70% vs. 19.67%) and drinkers (18.49% vs. 27.17% for light, 6.09% vs. 10.56% for moderate, and 4.48% vs. 6.99% for heavy-level drinking) in the PD group compared to the non-PD group.

**Table 1:** Baseline characteristics of study participants.

	Total (*N* = 3,931,741)	Parkinson Disease, no (*N* = 3,919,099)	Parkinson Disease, yes (*N* = 12,642)	Value of p^*^
Age (year), mean ± SD	55.1 ± 9.7	55.0 ± 9.6	66.1 ± 8.8	<0.001
Sex, male	2,029,800 (51.6)	2,023,607 (51.6)	6,193 (49.0)	<0.001
Body mass index (kg/m^2^)	24.0 ± 3.0	24.0 ± 3.0	24.1 ± 3.0	<0.001
Smoking intensity (*N*, %)				<0.001
None	696,075 (17.7)	693,898 (17.7)	2,177 (17.2)	
Light (<20 cigarettes/day)	2,546,417 (64.8)	2,536,955 (64.7)	9,462 (74.9)	
Moderate (20 cigarettes/day)	300,757 (7.7)	300,262 (7.7)	495 (3.9)	
Heavy (≥20 cigarettes/day)	388,492 (9.9)	387,984 (9.9)	508 (4.0)	
Smoking duration (year) (*N*, %)				<0.001
None	2,463,507 (62.7)	2,454,268 (62.6)	9,239 (73.1)	
< 5	58,794 (1.5)	58,652 (1.5)	142 (1.1)	
5–9	77,320 (2.0)	77,135 (2.0)	185 (1.5)	
10–19	32,6,061 (8.3)	325,422 (8.3)	639 (5.1)	
20–29	558,309 (14.2)	557,487 (14.2)	822 (6.5)	
≥ 30	447,750 (11.4)	446,135 (11.4)	1,615 (12.8)	
Smoking history (peak-year) (*N*,%)				<0.001
0	2,463,507 (62.7)	2,454,268 (62.6)	9,239 (73.1)	
< 10	364,988 (9.3)	364,115 (9.3)	873 (6.9)	
< 20	424,630 (10.8)	423,830 (10.8)	800 (6.3)	
< 30	338,029 (8.6)	337,334 (8.6)	695 (5.5)	
≥ 30	340,587 (8.7)	339,552 (8.7)	1,035 (8.2)	
Alcohol intake (*N*, %)				<0.001
None	2,175,517 (55.3)	2,166,548 (55.3)	8,969 (71.0)	
Light (<15 g/day)	1,067,096 (27.1)	1,064,759 (27.2)	2,337 (18.5)	
Moderate (≥15, <30 g/day)	414,704 (10.6)	413,934 (10.6)	770 (6.1)	
Heavy (≥ 30 g/day)	274,424 (7.0)	273,858 (7.0)	566 (4.5)	
Regular Exercise	868,848 (22.1)	866,226 (22.1)	2,622 (20.7)	<0.001
Smoking				<0.001
None	2,463,507 (62.7)	2,454,268 (62.6)	9,239 (73.1)	
Ex-smoker	696,075 (17.7)	693,898 (17.7)	2,177 (17.2)	
Current smoker	772,159 (19.6)	770,933 (19.7)	1,226 (9.7)	
Income level				<0.0001
1st Quartile	905,683 (23.0)	902,824 (23.0)	2,859 (22.6)	
2nd Quartile	708,957 (18.0)	706,986 (18.0)	1,971 (15.6)	
3rd Quartile	918,908 (23.4)	916,068 (23.4)	2,840 (22.5)	
4th Quartile	1,398,193 (35.6)	1,393,221 (35.6)	4,972 (39.3)	
Diabetes, yes	437,776 (11.1)	435,377 (11.1)	2,399 (19.0)	<0.0001
Dyslipidemia, yes	913,611 (23.2)	909,828 (23.2)	3,783 (29.9)	<0.0001

Data are presented as mean ± standard deviation for numerical variables and number (percentages) for categorical variables. N, number; SD: standard deviation. ^*^Student *t*-test for continuous variables (age and BMI) and *χ*^2^ test for categorical variables (all others).

### Risk of Parkinson’s disease by changes in cigarette smoking

Compared to the sustained non-smokers, sustained light smokers (adjusted hazard ratio [aHR] 0.80, 95% CI 0.75–0.85), sustained moderate smokers (aHR 0.54, 95% CI 0.47–0.61) and sustained heavy smokers (aHR 0.49, 95% CI 0.44–0.55) had lower risk of PD (Supplementary Table S2).

**Figure 2:**
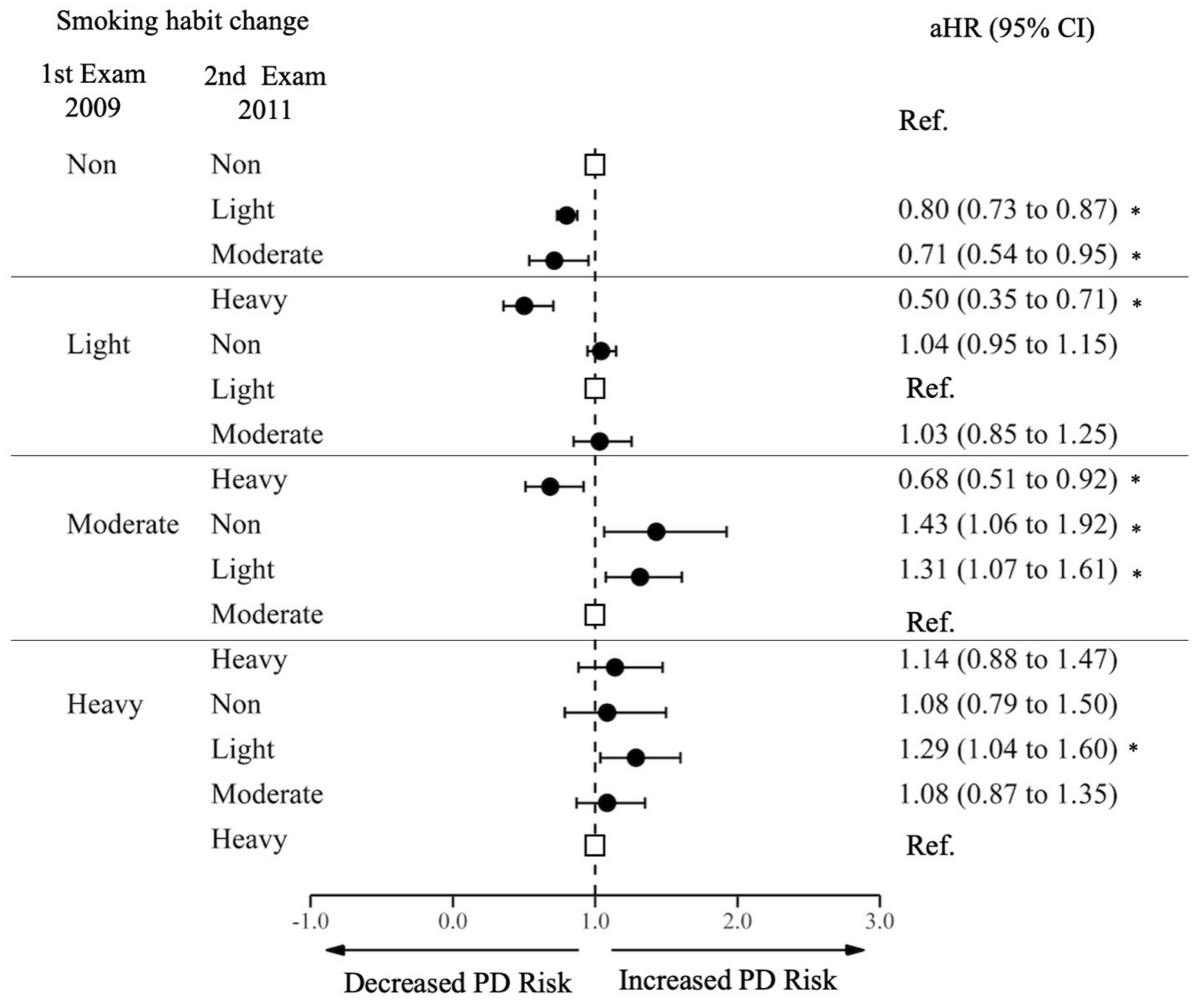
Risk of Parkinson’s Disease by changes in cigarette smoking. PD, Parkinson Disease; Ref., Reference; aHR, Adjusted Hazard Ratio; CI, Confidence Interval. The aHR was derived from the model, adjusting for factors including age, sex, income, baseline body mass index, baseline drinking status, and history of diabetes or dyslipidemia. An asterisk (*) indicates values that are statistically significant.

We analyzed the effect of changes in smoking habit on PD incidence (Figure 2; Supplementary Table S2). Among non-smokers at the first examination, those who initiated smoking to a light level (aHR 0.80 95% CI 0.73–0.87), moderate level (aHR 0.71 95% CI 0.54–0.95), and heavy level (aHR 0.50 95% CI 0.35–0.71) had a decreased risk of PD with a dose–response relationship when compared to those who continued to be non-smokers. Among light smokers at the first examination, those who increased frequency of smoking to a heavy level had a decreased risk of PD (aHR 0.68, 95% CI 0.51–0.92). Among moderate smokers at the first examination, those who reduced their frequency of smoking to light (aHR 1.32, 95% CI 1.08–1.61) or quit smoking (aHR 1.43, 95% CI 1.06–1.92) had an increased risk of PD. Among heavy smokers at the first examination, those who reduced their frequency of smoking to a light level had an increased risk of PD (aHR 1.29, 95% CI 1.04–1.60).

### Risk of Parkinson’s disease by changes in alcohol consumption

**Figure 3:**
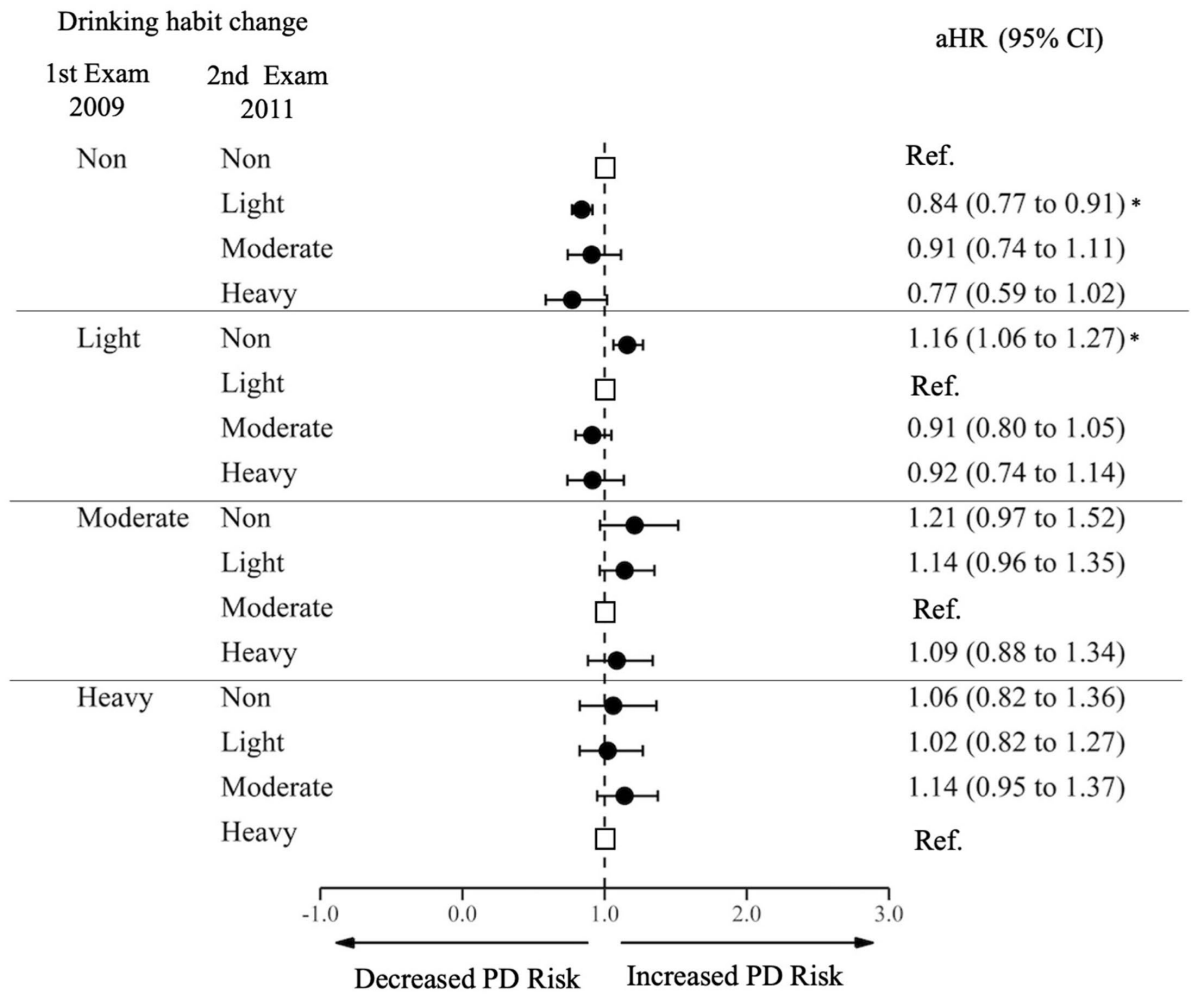
Risk of Parkinson’s Disease by changes in alcohol consumption. PD, Parkinson Disease; Ref., Reference; aHR, Adjusted Hazard Ratio; CI, Confidence Interval. The aHR was derived from the model, adjusting for factors including age, sex, income, baseline body mass index, baseline drinking status, and history of diabetes or dyslipidemia. An asterisk (*) indicates values that are statistically significant.

Compared to those who sustained non-drinking, sustained drinkers at all levels, sustained light drinkers (HR 0.85 95% CI 0.89–0.91), moderate sustainers (HR 0.68, 95% CI 0.60–0.78), and heavy sustainers (HR 0.77, 95% CI 0.68–0.87) had a decreased risk of PD. Among initial non-drinkers, those who began light drinking had a decreased risk of PD (aHR 0.84, 95% CI 0.77–0.91), and those who started heavy drinking showed even greater decrease in the risk (aHR 0.77, 95% CI 0.59–1.02), although the statistical significance was marginal (Figure 3; Supplementary Table S3). Among light drinkers at the first examination, those who stopped drinking had an increased risk of PD (aHR 1.16, 95% CI 1.06–1.23). Increasing or decreasing alcohol consumption between moderate and heavy drinking was not significantly associated with decreased or increased risk of PD.

### Risk of Parkinson’s disease by combined effect of changes in smoking and alcohol consumption

**Figure 4:**
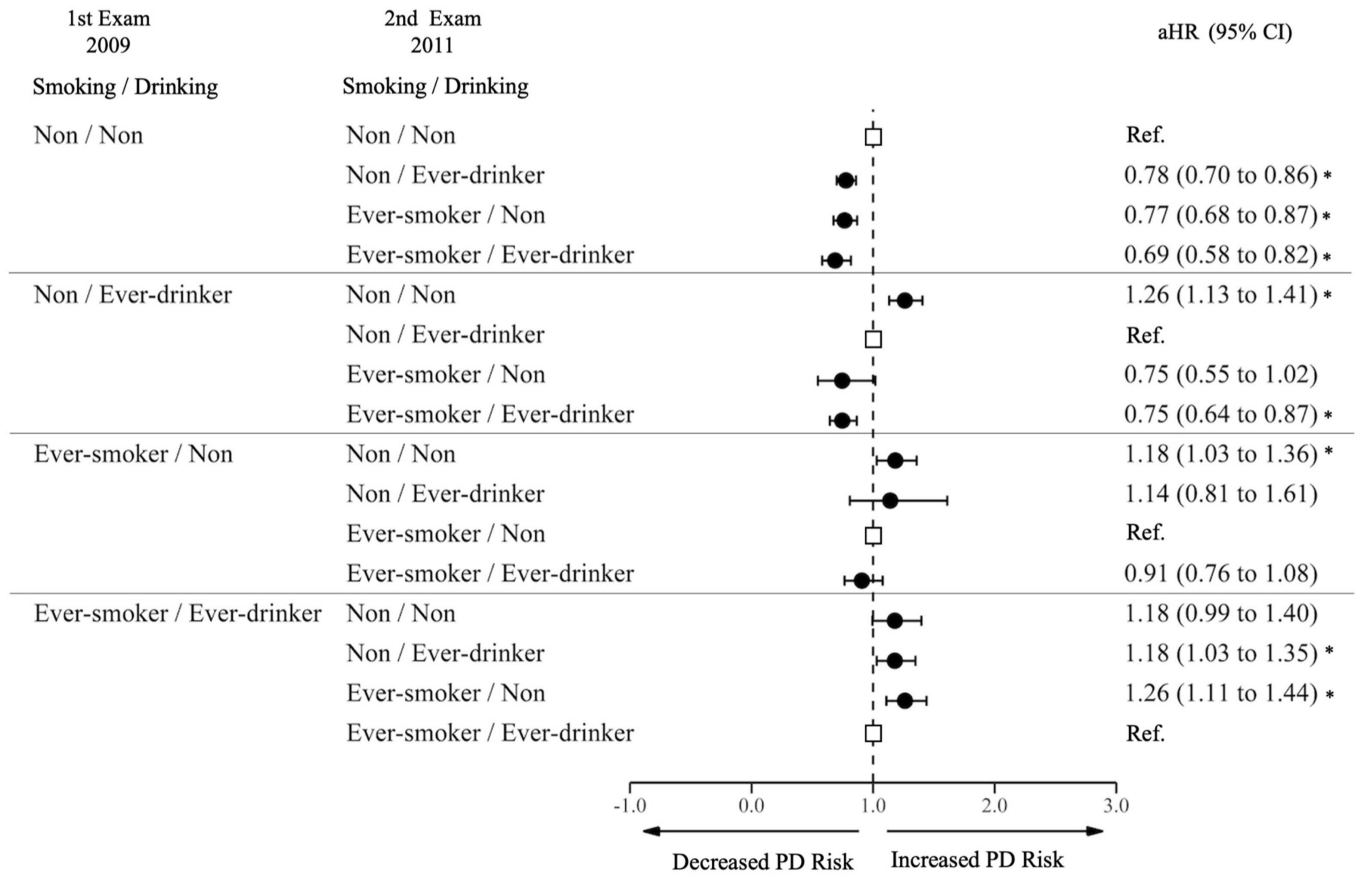
Risk of Parkinson’s disease by joint effect of changes in smoking and alcohol consumption. PD, Parkinson Disease; Ref., Reference; aHR, Adjusted Hazard Ratio; CI, Confidence Interval. The aHR was derived from the model, adjusting for factors including age, sex, income, baseline body mass index, baseline drinking status, and history of diabetes or dyslipidemia. An asterisk (*) indicates values that are statistically significant.

Among non-smoking and non-drinking participants at the first examination, those who initiated smoking only (aHR 0.78, 95% CI 0.70–0.86), drinking only (aHR 0.77, 95% CI 0.68–0.87), and both smoking and drinking (aHR 0.69, 95% CI 0.58–0.82) had decreased risks compared to those who continued to not smoke or drink. (Figure 4; Supplementary Table S4) Among non-smoking, drinking participants at the first examination, those who stopped drinking had an increased risk of PD (aHR 1.26, 95% CI 1.13–1.41); but those who sustained drinking and started smoking had a decreased risk of PD (aHR 0.75, 95% CI 0.64–0.87). Among smoking, drinking participants at the first examination, those who sustained smoking but stopped drinking had an increased risk of PD (aHR 1.26, 95% CI 1.11–1.44); and those who quit smoking, but sustained drinking had an increased risk of PD (aHR 1.18, 95% CI 1.03–1.35).

## Discussion

To the best of our knowledge, this is the first study to demonstrate the effects of combined changes in frequency of smoking and alcohol consumption on PD incidence with a large-scale nationwide cohort database. We also found that smoking and alcohol consumption at moderate levels are associated with decreased risk of PD development.

### Changes in smoking habit and PD risk

In our research, we found that sustained light smokers (aHR 0.80), sustained moderate smokers (aHR 0.54), and sustained heavy smokers (aHR 0.49) had lower risk of developing PD compared to sustained non-smokers. We also demonstrated that heavy smoking was associated with lower PD development risk with a clear dose–response relationship. In addition, we showed that smoking initiation or increased frequency of smoking was associated with decreased PD incidence, while smoking cessation or reduction was associated with increased risk of incident PD. Our results reinforce the conclusion of previous studies. The British study (Mappin-Kasirer et al., 2020) and another recent Mendelian randomization study (Dominguez-Baleon et al., 2021) reported that smoking has a protective effect on the risk of PD development. A previous meta-analysis of observational research reported that current smoking was associated with a 58% risk reduction for developing PD (Mappin-Kasirer et al., 2020). In a previous study based on a large cohort of 220,494 individuals, former smokers had a 20% risk reduction, and current smokers had a 50% risk reduction in developing PD compared with never smokers (Gallo et al., 2019). Furthermore, smoking and its interactions with genetic variants may affect PD risk (Chuang et al., 2017). In addition to the previous evidence, our study gives an additional information regarding the effect of smoking habit changes on PD risks.

Findings in this study shed light on potential protective mechanisms of smoking against PD. Building upon previous research, there is a proposition that an effective medication for PD could be formulated using chemical substances derived from tobacco (Barreto et al., 2014; Li et al., 2015). Nicotine, in particular, has been suggested to safeguard against the onset of PD by modulating dopamine levels. It enhances the activity of the dopamine circuit by facilitating dopamine release upon activation of the nicotine receptor (Bono et al., 2021). Notably, the potential therapeutic properties of nicotine in PD have not only been a subject of academic interest but also clinical investigations. For instance, a pilot trial titled “Transnasal Nicotine in Parkinson Disease” has been underway since 2019, as referenced by ClinicalTrials.gov (Identifier: NCT038651210). This trial is a testament to the ongoing exploration of the efficacy of tobacco-derived components, particularly nicotine, in treating PD. The outcomes of such studies, combined with our findings, underscore the potential of tobacco chemicals as candidates for PD treatment.

### Changes of alcohol consumption and PD risk

Our study revealed that light sustained drinkers, moderate sustainers, and heavy sustainers showed a decreased risk of PD compared to those who sustained non-drinking. We also showed that those who started drinking at a light level (less than 15 g/day on average) had decreased risk of PD. This result is similar to findings in a previous meta-analysis of 11 prospective studies demonstrating that alcohol consumption was associated with decreased risk of PD. The association was nearly U-shaped (Shao et al., 2021). The risk of PD decreased (smallest RR 0.81) with the amount of daily drinking. Consumption of 26 g/day, 26–36 g/day (lowest), and 36 g/day demonstrated a U-shaped association. Consumption of 26–36 g/day was considered to be a moderate to heavy level of drinking in our research. Therefore, the quantity of alcohol consumed is important for decreasing PD risk; however, the optimal amount may differ by race and ethnicity.

In addition, we showed that initiation of alcohol consumption was associated with decreased future risk of PD, and drinking cessation was associated with increased risk of PD. Although a similar trend was observed, the increase or decrease in alcohol consumption did not reach statistical significance probably because of a smaller effect than that of the smoking and a U-shaped association. Furthermore, an increase of alcohol consumption to heavy drinking or reduction of alcohol consumption from heavy drinking was not associated with altered risk, further suggesting a U-shaped risk of PD with level of alcohol consumption (Belvisi et al., 2020). Our result on change in alcohol consumption and PD risk further strengthens the evidence found in previous studies that found light to moderate alcohol consumption may have protective effects on PD incidence, while heavy drinking does not.

Alcohol acts directly on the brain, increasing oxidative stress and inducing inflammatory reactions (Ascherio and Schwarzschild, 2016). Thus, alcohol use disorder is associated with various neurodegenerative diseases (Kamal et al., 2020; Peng et al., 2020). Chronic severe alcohol consumption induces excitotoxicity and oxidative stress caused by glutamic acid, resulting in permanent neuronal disorders. Furthermore, alcohol consumption can have direct interactions with the gut barrier, contributing to inflammation. This is noteworthy, especially considering recent findings that link gut inflammation to Parkinson’s disease (Gorecki et al., 2019). Regarding the U-shaped association, biological components of alcohol may influence the risk of PD. For example, a moderate amount of the urate-raising effects of beer (Gibson et al., 1984; Gaffo et al., 2010),and the flavonoids present in red wine (Commenges et al., 2000) may protect against PD. Purin, which is abundant in beer, works as a free radical scavenger; flavonoids, which are abundant in red wine, have a neuroprotective role. Therefore, drinking a modest amount of alcohol can be beneficial (Liu et al., 2013). In our results, moderate alcohol consumption seemed to have a more profound protective effect than a neurotoxic effect. PD risk was reduced in non-drinkers-turned-light drinkers, and light drinkers had increased risk when ceasing alcohol consumption. Likewise, light, moderate, and heavy drinking sustainers had decreased risk of PD compared to non-drinking sustainers.

### Combined effect of changes in smoking and alcohol consumption

The novel findings of our research involve the combined effect of smoking frequency and alcohol consumption on PD risk. In a previous study based on Korean NHIS data, the authors found that current smoking and alcohol consumption independently reduced the risk of PD in both men and women. In some populations, such as current male smokers and female ex-smokers, the authors discovered a super-additive interaction effect between smoking and alcohol use (Kim et al., 2020a). However, this research group did not measure combined effects of changes in smoking frequency and alcohol consumption. In our study, among non-smokers non-drinkers at the time of the first examination, those who started both smoking and drinking had a greater reduction in PD risk than those who started only one of the two. Conversely, those smokers and drinkers at the time of the first examination who subsequently ceased both smoking and drinking had a greater increase in PD risk than those who ceased one of the two habits. This finding suggests that smoking and alcohol consumption may have a joint protective effect against of PD. A systematic review suggested that smoking may modify the association between alcohol consumption and PD risk (Bettiol et al., 2015). Further research is needed to elucidate the mechanism by which smoking, and alcohol may interact in reducing future PD.

Our study has several potential limitations. First, our analysis focused on individuals who participated in national health examinations in both 2009 and 2011. This may imply that our study population was generally healthier than the general population. Second, self-reported data on smoking and alcohol consumption might be underestimated, leading to a subdued inverse relationship in our findings. Third, our study did not account for the age at which individuals began cigarette smoking or alcohol consumption, as well as potential changes in smoking and drinking habit after the year 2011. Additionally, we did not factor in potential changes in smoking and drinking habits after the year 2011, which might have influenced the observed associations. Fourth, our study did not account for the duration of smoking and drinking habits, which could offer a more comprehensive perspective. Fifth, the estimation of PD incidence might not be accurate due to the challenges in identifying PD based on prolonged prodromal symptoms before an official diagnosis. However, it’s worth noting that in South Korea, patients with apparent clinical symptoms of PD are typically diagnosed and get medical care easily, given the national universal health coverage. Sixth, patients with cancer, stroke, and MI were excluded from our study. While these conditions were excluded based on their known associations with PD, they also represent some of the negative health consequences of smoking and drinking. This could potentially omit certain aspects of the relationship between smoking, drinking, and PD risk. Lastly, the smoking prevalence among South Korean women is notably lower than in Western countries. The smoking and drinking habits of South Koreans might also differ from other populations, potentially affecting the generalizability of our results. While the duration and quantity of alcohol consumption are crucial in establishing a cause-effect relationship, the frequency of heavy drinking episodes also emerges as a significant risk factor, warranting future research (Room, 2000). Moreover, it’s important to note that our study primarily focused on the general Parkinson’s disease onset and did not differentiate between the different types of PD, such as autosomal recessive juvenile PD (AR-JP). Considering that AR-JP patients represent only about 10–15% of the PD population, we believe that its influence on our overall results might not be significant. However, in future research, it might be beneficial to conduct studies excluding the AR-JP subtype to ensure a more comprehensive understanding of the relationship between tobacco, alcohol, and PD onset.

We confirmed the presence of a dose–response protective effect of smoking and possibly alcohol consumption against the risk of PD. In addition, we also demonstrated that smoking and drinking habit changes can influence the incidence of PD. Further studies are warranted to find the possible mechanisms for the protective effects of smoking and drinking on PD, which may present insights into the etiology of PD.

## Data availability statement

The data analyzed in this study is subject to the following licenses/restrictions: Data from the National Health Insurance Service (NHIS) of South Korea can be accessed from the website (http://nhiss.nhis.or.kr). However, researchers should submit a research proposal to acquire the approval with an appropriate IRB document. Requests to access these datasets should be directed to KH, hkd917@naver.com.

## Ethics statement

The studies involving humans were approved by the Institutional Review Board of the Samsung Medical Center (IRB No. SMC-2020-12-108). The studies were conducted in accordance with the local legislation and institutional requirements. Written informed consent for participation was not required from the participants or the participants’ legal guardians/next of kin in accordance with the national legislation and institutional requirements.

## Author contributions

SYJ drafted the manuscript. DS and SC supervised the entire process. EC, KH, JY, YY, JEY, J-HM, and SMJ contributed to interpretation of data. All authors contributed to the article and approved the submitted version.

## Funding

J-HM received a grant from the National Research Foundation of Korea and SMC Research and Development Grant.

## Conflict of interest

J-HM has lectured, consulted, and received Honoria from Bayer Schering Pharma, Merck Serono, Biogen Idec, Sanofi Genzyme, Teva-Handok, UCB, Samsung Bioepis, Mitsubishi Tanabe Pharma, and Roche.

The remaining authors declare that the research was conducted in the absence of any commercial or financial relationships that could be construed as a potential conflict of interest.

## Publisher’s note

All claims expressed in this article are solely those of the authors and do not necessarily represent those of their affiliated organizations, or those of the publisher, the editors and the reviewers. Any product that may be evaluated in this article, or claim that may be made by its manufacturer, is not guaranteed or endorsed by the publisher.
