# Supplementary material for: Changes in smoking, alcohol consumption, and the risk of Parkinson’s disease
Source: Front Aging Neurosci. 2023 Sep 13;15:1223310. doi: 10.3389/fnagi.2023.1223310 (PMC10525683; doi:10.3389/fnagi.2023.1223310)
Supplement: Supplementary file 1 [file Table_1.docx]

**Supplementary Table 1.** Previous Studies

| Author | year | Country | Disease (Exposure variables) | Study design | Study setting | Number of participants | Main finding |
| --- | --- | --- | --- | --- | --- | --- | --- |
| Meta-Analysis and Review | | | | | | | |
| Zhang et al. | 2014 | China | Alcohol | Meta Analysis | Population-Based | 32 articles, 677,550 subjects (9994 cases) | Smoking-adjusted RR: 0.78 (95% CI 0.67-0.92), 0.86 for prospective studies, 0.74 for matched case-control studies.  The risk of PD decreased by 5% for every 1drink/day increment in alcohol intake (95% CI 0.89-1.02) in a linear (Pfor nonlinearity = 0.85) dose-response manner |
| Bettiol et al. | 2015 | Australia | Alcohol | Review | Population-Based | 16 articles | 7 Case-control studies : a weak protective association of alcohol reported than prospective studies Smoking may modify the association between alcohol intake and PD risk |
| Li et al. | 2015 | China | Smoking | Meta Analysis | Population-Based | 61 case-control and 8 cohort studies | RR, 0.59 (95% CI, 0.56-0.62) for ever smokers compared with never smokers. The summary RR for those smoking more than 30 pack-years was 0.39 (95% CI, 0.29-0.53)and 0.66 (95% CI, 0.49-0.88) for those smoking less than 30 pack-years: **inverse association between cigarette smoking and the risk of PD.** |
| Shao et al. | 2021 | China | Alcohol | Meta Analysis | Population-Based | 11 prospective studies included | RR, 0.81 (95% CI 0.70-0.95) for higher intake of alcohol. RR 0.78 (95% CI 0.65-0.94) for beer intaker. RR 0.66, 95% CI 0.55-0.80) for studies in Asia |
| Peng et al. | 2020 | US | Alcohol | Review | Population-Based | 8 Studies | Alcohol has been suggested to be either protective of, or not associated with, PD. However, experimental animal studies indicate that chronic heavy alcohol consumption may have dopamine neurotoxic effects through the induction of Cytochrome P450 2E1 (CYP2E1) and an increase in the amount of α-Synuclein (αSYN) relevant to PD. |
| Belvisi et al. | 2019 | Italy | Alcohol, Smoking | Review | Population-Based | Not Available | Six studies assessed the relationship between alcohol intake and PD. Four studies did not ﬁnd any signiﬁcant association between exposure and outcome (Grandinetti et al., 1994; Hernán et al., 2003; Tan et al., 2008; Palacios et al., 2012) whereas two suggested that alcohol may exert some degree of protection (Paganini-Hill, 2001; Liu et al., 2013) (Table 2, supplementary material). These contrasting results do not appear to be due to diﬀerences between studies in factors that contribute to study power. **Although the power of one of the studies in which no association between alcohol intake and PD, which enrolled 8000 subjects but only yielded 58 PD patients over a 26-year-follow-up period (Grandinetti et al., 1994) was probably low, all the other studies in which no association was reported were characterized by large study cohorts (63,257 to 177,229 participants) and identiﬁed large numbers of PD patients (157 to 605)**  Indeed, there appears to be an inverse association between PD and the intensity and duration of smoking, which is more pronounced in current than in former smokers, decreases as the number of years after smoking has ceased increases, and is observed with a range of tobacco products (Grandinetti et al., 1994; Hernán et al., 2001; Paganini-Hill, 2001; Thacker et al., 2007; Tan et al., 2008; Sääksjärvi et al., 2008; Chen et al., 2009, 2010; Kyrozis et al., 2013) (Table 2, supplementary material). **The inverse relationship between smoking and PD may depend either on the eﬀect of nicotine on the central nervous system (Quik et al., 2012) or alternatively may reﬂect an inverse causality due to a premorbid personality trait making PD parients less inclined to start or continue to smoke (Evans et al., 2006).** |
| Original Investigation | | | | | | | |
| Gallo et al. | 2019 | UK, Netherlands, Germany, Spain, Italy and Greece | Smoking | Cohort | Population-Based | 220,494 subjects | HR: smoking <20 years were 0.84 [95% confidence interval (CI) 0.67-1.07], 20-29 years 0.73 (95% CI 0.56-0.96) and >30 years 0.54 (95% CI 0.43-0.36) compared with never smokers. |
| Kim et al. | 2020 | Korea | Smoking | Cohort | Population-Based | 3,400,538 men and 3,395,278 women | HR: 0.50 (95% CI 0.56-0.96) for current male smokers, 0.77 (95% CI 0.68-0.72) compared to nonsmokers 0.83 (95% CI 0.80-0.86) for male ex-smokers A superadditive interaction: Alcohol and Current smoking in male (RERI, 0.19; 95% CI, 0.04 to 0.34; P = 0.015) and female (RERI, 0.42; 95% CI, 0.09 to 0.76; P = 0.014) |
| Mappin-Kasirer et al. | 2020 | UK | Smoking | Cohort | Population-Based | 30,000 male doctors | After adjustment for age at risk, current smokers at baseline had a 30% lower risk of PD (RR 0.71; 95% CI 0.60–0.84), and continuing smokers classiﬁed using updated smoking habits at resurvey had a 40% lower risk (RR 0.60; 95% CI 0.46–0.77) |
| Peters et al. | 2020 | Italy, Spain, UK, Netherlands, Greece, Germany, Sweden | Alcohol | Cohort | Population-Based | 521,000 | **No associations between baseline or lifetime total alcohol consumption and PD risk** Men with moderate lifetime consumption (5-29.9 g/day) were at ~50% higher risk compared with light consumption (0.1-4.9 g/day) HR 1.52 (95% CI 1.00–2.33) |
| Dominguez-Baleon et al. | 2021 | US | Alcohol, Smoking | Cross-sectional Mendelian randomization | Population-Based | 1.2 million participants | HR: Alcohol OR 0.79 (95% CI 0.65–0.96), Smoking (current vs. former) OR 0.64 (95% CI 0.46–0.89)  Our findings support the role of smoking as a protective factor against PD, but only when comparing current vs. former smokers. Similarly, s, with the alcohol dehydrogenase 1B (ADH1B) locus as a potential candidate for further investigation of the mechanisms underlying this association. |
| Yoon et al | 2022 | Korea | Smoking, Alcohol, Physical activity | Cohort | Population-Based | Case 2665  Control 453,274 | Current smoker, OR 0.556, 95% CI 0.488–0.632, ex-smoker, odds ratio [OR] 0.782, 95% conﬁdence interval [CI] 0.713–0.858,  Alcohol consumption≤3/week, OR 0.717, 95% CI 0.658–0.780 for men, OR 0.674, 95% CI 0.579–0.785 for women  Physical activity at moderate level, OR 0.894, 95% CI 0.837–0.955 for men, OR 0.792, 95% CI 0.748–0.840 for women |
| Kim et al. | 2020 | Korea | Smoking, Weight change | Cohort | Population-Based | Case 6,871 | The overall risk of PD was significantly lower in quitters than in never smokers [hazard ratio (HR)=0.78, 95% confidence interval (CI)=0.70–0.86]. The risk of PD was still lower in quitters with BMI increase (HR=0.80, 95% CI=0.65–0.98) and in those with BMI maintenance (HR=0.77, 95% CI=0.68–0.87). This tendency was also observed in quitters with BMI decrease (HR=0.76, 95% CI=0.55–1.06). BMI increase (HR=1.10, 95% CI=1.02–1.18) but not BMI decrease (HR=1.06, 95% CI=0.98–1.14) significantly increased the PD risk compared to BMI maintenance. |
| Kim et al. | 2020 | Korea | Alcohol | Cohort | Population-Based | 1,309,267 women | There were no significant trends in alcohol-related PD risk among never smokers. Additionally, examining this association by type of alcohol intake also yielded null findings.These results do not support an association between alcohol intake and PD risk in women. |

**Supplementary Table 2. Smoking change and Parkinson’s disease incidence**

| Smoking status at 1^st^ exam | Smoking status at 2^nd^ exam | Total | Incidence | Person-years | IR | Sustained non-smokers as the reference | | | Smoking status at the 1^st^ exam as the reference | | |
| --- | --- | --- | --- | --- | --- | --- | --- | --- | --- | --- | --- |
|  |  |  |  |  |  | Model 1 | Model 2 (aHR) | Model 3 (aHR) | Model 1 | Model 2 (aHR) | Model 3 (aHR) |
| Non | Non | 2,278,233 | 8,509 | 14,520,919.20 | 0.59 | 1 (Ref.) | 1 (Ref.) | 1 (Ref.) | 1(Ref.) | 1(Ref.) | 1(Ref.) |
|  | Light | 145,387 | 621 | 915,630.77 | 0.68 | **1.16 (1.07,1.26)** | **0.77 (0.71,0.85)** | **0.80 (0.73,0.87)** | **1.16 (1.07,1.26)** | **0.77 (0.71,0.84)** | **0.80 (0.73,0.87)** |
|  | Moderate | 21,661 | 47 | 136,008.36 | 0.35 | **0.59 (0.45,0.79)** | **0.69 (0.52,0.92)** | **0.72 (0.54,0.96)** | **0.59 (0.45,0.79)** | **0.68 (0.51,0.90)** | **0.71 (0.54,0.95)** |
|  | Heavy | 23,318 | 33 | 146,549.14 | 0.23 | **0.39 (0.28,0.55)** | **0.49 (0.35,0.69)** | **0.51 (0.36,0.72)** | **0.39 (0.28,0.55)** | **0.48 (0.34,0.68)** | **0.50 (0.36,0.71)** |
| Light | Non | 143,849 | 627 | 905,976.95 | 0.69 | **1.19 (1.09,1.29)** | **0.83 (0.76,0.90)** | **0.83 (0.76,0.91)** | **1.49 (1.36,1.64)** | 1.06 (0.96,1.16) | 1.04 (0.95,1.15) |
|  | Light | 514,338 | 1,505 | 3,251,633.30 | 0.46 | **0.79 (0.75,0.84)** | **0.77 (0.72,0.82)** | **0.80 (0.75,0.85)** | 1(Ref.) | 1(Ref.) | 1(Ref.) |
|  | Moderate | 45,257 | 108 | 284,710.60 | 0.38 | **0.65 (0.54,0.79)** | **0.77 (0.64,0.94)** | **0.81 (0.67,0.98)** | 0.82 (0.68,1.00) | 1.01 (0.83,1.22) | 1.03 (0.85,1.26) |
|  | Heavy | 31,575 | 46 | 198,449.56 | 0.23 | **0.40 (0.30,0.53)** | **0.51 (0.38,0.68)** | **0.52 (0.39,0.70)** | **0.50 (0.37,0.67)** | **0.67 (0.50,0.89)** | **0.68 (0.51,0.92)** |
| Moderate | Non | 19,938 | 59 | 125,115.18 | 0.47 | **0.81 (0.63,1.05)** | **0.77 (0.59,0.99)** | **0.76 (0.59,0.98)** | **2.08 (1.56,2.76)** | **1.47 (1.10,1.97)** | **1.43 (1.06,1.92)** |
|  | Light | 66,237 | 164 | 416,714.36 | 0.39 | **0.68 (0.58,0.79)** | **0.69 (0.59,0.81)** | **0.72 (0.62,0.85)** | **1.74 (1.42,2.12)** | **1.33 (1.09,1.63)** | **1.32 (1.08,1.61)** |
|  | Moderate | 164,564 | 234 | 1,034,838.80 | 0.23 | **0.39 (0.34,0.44)** | **0.51 (0.44,0.58)** | **0.54 (0.47,0.61)** | 1(Ref.) | 1(Ref.) | 1(Ref.) |
|  | Heavy | 49,251 | 79 | 308,744.71 | 0.26 | **0.44 (0.35,0.55)** | **0.58 (0.47,0.73)** | **0.61 (0.49,0.77)** | 1.13 (0.88,1.46) | 1.16 (0.90,1.49) | 1.14 (0.88,1.47) |
| Heavy | Non | 21,487 | 44 | 134,638.49 | 0.33 | **0.56 (0.42,0.75)** | **0.58 (0.43,0.78)** | **0.56 (0.41,0.75)** | **1.66 (1.21,2.27)** | 1.12 (0.82,1.54) | 1.09 (0.79,1.50) |
|  | Light | 53,030 | 110 | 334,170.52 | 0.33 | **0.57 (0.47,0.68)** | **0.63 (0.52,0.76)** | **0.65 (0.53,0.78)** | **1.67 (1.35,2.07)** | **1.27 (1.03,1.58)** | **1.29 (1.04,1.60)** |
|  | Moderate | 69,272 | 106 | 434,015.66 | 0.24 | **0.42 (0.35,0.51)** | **0.52 (0.43,0.63)** | **0.54 (0.44,0.66)** | **1.25 (1.00,1.55)** | 1.05 (0.84,1.30) | 1.08 (0.87,1.35) |
|  | Heavy | 284,344 | 350 | 1,783,713.16 | 0.20 | **0.34 (0.30,0.38)** | **0.47 (0.42,0.53)** | **0.49 (0.44,0.55)** | 1(Ref.) | 1(Ref.) | 1(Ref.) |

Data are presented as mean ± standard deviation for numerical variables and number (percentages) for categorical variables.

N, number; Ref.,Reference, IR, Incidence Rate

Model 1: Unadjusted, Model 2: Adjusted for age and sex, Model 3: Adjusted for age, sex, income, body mass index, and drinking status at baseline, and history of diabetes or dyslipidemia.

Statistically signiﬁcant values are marked in bold

**Supplementary Table 3. Alcohol consumption habit change and Parkinson’s disease incidence**

| Alcohol consumption at 1^st^ exam | Alcohol consumption at 2^nd^ exam | Total | Incidence | Person-years | IR | Sustained non-drinkers as the reference | | | Drinking status at the 1^st^ exam as the reference | | |
| --- | --- | --- | --- | --- | --- | --- | --- | --- | --- | --- | --- |
|  |  |  |  |  |  | Model 1 | Model 2 (aHR) | Model 3 (aHR) | Model 1 | Model 2 (aHR) | Model 3 (aHR) |
| Non | Non | 1,863,033 | 7,887 | 11,842,767.87 | 0.67 | 1(Ref.) | 1(Ref.) | 1(Ref.) | 1(Ref.) | 1(Ref.) | 1(Ref.) |
|  | Light | 243,690 | 595 | 1,545,812.06 | 0.38 | **0.58(0.53,0.63)** | **0.83(0.76,0.90)** | **0.85(0.78,0.93)** | **0.58(0.53,0.63)** | **0.82(0.75,0.89)** | **0.84(0.77,0.91)** |
|  | Moderate | 34,686 | 95 | 218,976.98 | 0.43 | **0.65(0.54,0.80)** | 0.87(0.71,1.06) | 0.92(0.75,1.13) | **0.65(0.53,0.80)** | 0.86(0.70,1.05) | 0.91(0.74,1.12) |
|  | Heavy | 20,386 | 52 | 128,192.60 | 0.41 | **0.61(0.47,0.80)** | **0.73(0.56,0.96)** | 0.78(0.59,1.02) | **0.61(0.47,0.80)** | **0.73(0.55,0.95)** | 0.77(0.59,1.02) |
| Light | Non | 253,563 | 876 | 1,607,715.06 | 0.54 | **0.82(0.77,0.88)** | 1.01(0.94,1.09) | 1.01(0.94,1.09) | **1.63(1.50,1.77)** | **1.21(1.11,1.32)** | **1.16(1.06,1.27)** |
|  | Light | 623,430 | 1,319 | 3,953,122.67 | 0.33 | **0.50(0.48,0.53)** | **0.82(0.77,0.87)** | **0.85(0.80,0.91)** | 1(Ref.) | 1(Ref.) | 1(Ref.) |
|  | Moderate | 129,502 | 245 | 819,661.44 | 0.30 | **0.45(0.40,0.51)** | **0.72(0.63,0.82)** | **0.78(0.68,0.89)** | 0.90(0.78,1.03) | 0.88(0.77,1.01) | 0.91(0.80,1.05) |
|  | Heavy | 38,914 | 89 | 245,657.01 | 0.36 | **0.55(0.44,0.67)** | **0.73(0.59,0.90)** | **0.79(0.64,0.98)** | 1.08(0.88,1.34) | 0.88(0.71,1.09) | 0.92(0.74,1.14) |
| Moderate | Non | 36,295 | 123 | 228,458.07 | 0.54 | **0.81(0.68,0.97)** | 0.90(0.75,1.07) | 0.88(0.74,1.05) | **2.28(1.84,2.83)** | **1.36(1.09,1.70)** | 1.21(0.97,1.52) |
|  | Light | 150,517 | 305 | 951,391.37 | 0.32 | **0.48(0.43,0.54)** | **0.74(0.66,0.84)** | **0.80(0.71,0.90)** | **1.36(1.15,1.61)** | 1.17(0.99,1.38) | 1.14(0.97,1.35) |
|  | Moderate | 167,612 | 249 | 1,059,641.32 | 0.23 | **0.36(0.31,0.40)** | **0.63(0.55,0.71)** | **0.68(0.6,0.78)** | 1(Ref.) | 1(Ref.) | 1(Ref.) |
|  | Heavy | 74,791 | 138 | 472,159.64 | 0.29 | **0.44(0.37,0.52)** | **0.70(0.59,0.83)** | **0.76(0.64,0.91)** | **1.24(1.01,1.53)** | 1.09(0.89,1.34) | 1.09(0.88,1.34) |
| Heavy | Non | 22,626 | 83 | 140,931.06 | 0.59 | 0.89(0.71,1.10) | 0.87(0.70,1.08) | 0.84(0.68,1.05) | **1.81(1.42,2.31)** | 1.16(0.91,1.49) | 1.06(0.83,1.37) |
|  | Light | 49,459 | 118 | 311,503.08 | 0.38 | **0.57(0.48,0.69)** | **0.75(0.63,0.90)** | **0.80(0.67,0.97)** | 1.17(0.94,1.45) | 1.04(0.84,1.29) | 1.02(0.82,1.27) |
|  | Moderate | 82,904 | 181 | 522,164.73 | 0.35 | **0.52(0.45,0.61)** | **0.80(0.69,0.93)** | 0.88(0.75,1.02) | 1.07(0.89,1.29) | 1.15(0.95,1.38) | 1.14(0.95,1.37) |
|  | Heavy | 140,333 | 287 | 883,673.75 | 0.32 | **0.49(0.44,0.55)** | **0.70(0.62,0.80)** | **0.77(0.68,0.87)** | 1(Ref.) | 1(Ref.) | 1(Ref.) |

Data are presented as mean ± standard deviation for numerical variables and number (percentages) for categorical variables.

N, number; Ref.,Reference; IR, Incidence Rate

Model 1: Unadjusted, Model 2: Adjusted for age and sex, Model 3: Adjusted for age, sex, income, body mass index, and drinking status at baseline, and history of diabetes or dyslipidemia.

Statistically signiﬁcant values are marked in bold.

**Supplementary Table 4. Smoking and alcohol consumption habit change and Parkinson’s disease incidence**

| At initial | At 2-year follow-up | Total | Incidence | Person-years | IR | Sustained non-drinkers as the reference | | | Drinking status at the first examination as the reference | | |
| --- | --- | --- | --- | --- | --- | --- | --- | --- | --- | --- | --- |
|  |  |  |  |  |  | Model 1 | Model 2 (aHR) | Model 3 (aHR) | Model 1 | Model 2 (aHR) | Model 3 (aHR) |
| Smoking/Drinking | Smoking/Drinking |  |  |  |  |  |  |  |  |  |  |
| Non / Non | Non/Non | 1,525,001 | 6,544 | 9,726,994.40 | 0.67 | 1(Ref.) | 1(Ref.) | 1(Ref.) | 1(Ref.) | 1(Ref.) | 1(Ref.) |
|  | Non/Ever | 170,400 | 403 | 1,085,052.30 | 0.37 | **0.55 (0.50,0.61)** | **0.79 (0.71,0.87)** | **0.80 (0.72,0.88)** | **0.55 (0.50,0.61)** | **0.77 (0.70,0.85)** | **0.78 (0.70,0.86)** |
|  | Ever / Non | 53,296 | 289 | 333,135.36 | 0.87 | **1.30 (1.15,1.46)** | **0.75 (0.67,0.85)** | **0.76 (0.67,0.86)** | **1.29 (1.15,1.46)** | **0.77 (0.68,0.87)** | **0.77 (0.68,0.87)** |
|  | Ever/ Ever | 50,682 | 145 | 319,634.58 | 0.45 | **0.68 (0.58,0.80)** | **0.71 (0.60,0.84)** | **0.71 (0.60,0.84)** | **0.68 (0.58,0.80)** | **0.70 (0.59,0.83)** | **0.69 (0.58,0.82)** |
| Non/ Ever | Non/Non | 176,240 | 612 | 1,122,911.79 | 0.55 | **0.81 (0.75,0.88)** | 1.00 (0.92,1.08) | 1.00 (0.92,1.09) | **1.48 (1.34,1.64)** | **1.28 (1.15,1.42)** | **1.26 (1.13,1.41)** |
|  | Non/Ever | 406,592 | 950 | 2,585,960.67 | 0.37 | **0.55 (0.51,0.59)** | **0.79 (0.73,0.85)** | **0.79 (0.74,0.86)** | 1(Ref.) | 1(Ref.) | 1(Ref.) |
|  | Ever / Non | 10,053 | 42 | 62,859.10 | 0.67 | 1.00 (0.74,1.35) | **0.62 (0.46,0.84)** | **0.62 (0.45,0.84)** | **1.82 (1.33,2.48)** | 0.76 (0.55,1.03) | 0.75 (0.55,1.02) |
|  | Ever/ Ever | 76,335 | 225 | 482,559.24 | 0.47 | **0.70 (0.61,0.80)** | **0.61 (0.53,0.70)** | **0.61 (0.53,0.70)** | **1.27 (1.10,1.47)** | **0.75 (0.65,0.87)** | **0.75 (0.64,0.87)** |
| Ever / Non | Non/Non | 54,491 | 290 | 340,913.97 | 0.85 | **1.27 (1.13,1.43)** | **0.78 (0.69,0.88)** | **0.78 (0.69,0.88)** | **1.60 (1.40,1.83)** | **1.19 (1.04,1.37)** | **1.18 (1.03,1.36)** |
|  | Non/Ever | 8,438 | 34 | 53,028.90 | 0.64 | 0.96 (0.68,1.34) | 0.75 (0.53,1.05) | 0.74 (0.53,1.05) | 1.21 (0.86,1.70) | 1.16 (0.82,1.63) | 1.14 (0.81,1.61) |
|  | Ever / Non | 230,245 | 764 | 1,441,724.13 | 0.53 | **0.79 (0.74,0.86)** | **0.65 (0.59,0.70)** | **0.65 (0.60,0.70)** | 1(Ref.) | 1(Ref.) | 1(Ref.) |
|  | Ever/ Ever | 69,242 | 160 | 435,265.86 | 0.37 | **0.55 (0.47,0.64)** | **0.59 (0.50,0.69)** | **0.59 (0.50,0.69)** | **0.69 (0.59,0.82)** | 0.91 (0.77,1.08) | 0.91 (0.77,1.08) |
| Ever / Ever | Non/Non | 43,754 | 153 | 275,131.32 | 0.56 | **0.83 (0.71,0.98)** | 0.72 (0.61,0.85) | **0.71 (0.61,0.84)** | **2.09 (1.77,2.46)** | 1.19 (1.00,1.40) | 1.18 (1.00,1.40) |
|  | Non/Ever | 78,591 | 253 | 496,656.42 | 0.51 | **0.76 (0.67,0.86)** | **0.70 (0.62,0.80)** | **0.70 (0.61,0.80)** | **1.91 (1.67,2.18)** | **1.19 (1.04,1.36)** | **1.18 (1.03,1.35)** |
|  | Ever / Non | 82,437 | 275 | 516,201.98 | 0.53 | **0.80 (0.71,0.90)** | **0.75 (0.66,0.85)** | **0.74 (0.66,0.85)** | **2.00 (1.76,2.28)** | **1.26 (1.11,1.44)** | **1.26 (1.11,1.44)** |
|  | Ever/ Ever | 895,944 | 1,503 | 5,653,798.70 | 0.27 | **0.40 (0.38,0.42)** | **0.55 (0.51,0.59)** | **0.55 (0.51,0.59)** | 1(Ref.) | 1(Ref.) | 1(Ref.) |

Data are presented as mean ± standard deviation for numerical variables and number (percentages) for categorical variables.

N, number; Ref.,Reference; IR, Incidence Rate

Model 1: Unadjusted, Model 2: Adjusted for age and sex, Model 3: Adjusted for age, sex, income, body mass index, and drinking status at baseline, and history of diabetes or dyslipidemia.

Statistically signiﬁcant values are marked in bold.
